# Nosocomial oral myiasis caused by *Chrysomya bezziana* in Iran: A new case and review of human myiasis in the country

**DOI:** 10.1016/j.idcr.2026.e02497

**Published:** 2026-01-19

**Authors:** Seyed Reza Mirbadie, Mohammad Ali Mohaghegh, Fateme Skandary, Eissa Soleymani, Fatemeh Nikbin, Alireza Sazmand

**Affiliations:** a School of Medicine, Shahroud University of Medical Sciences, Shahroud, Iran; b Department of Laboratory Sciences, School of Paramedical Sciences, Torbat Heydariyeh University of Medical Sciences, Torbat Heydariyeh, Iran; c Health Sciences Research Center, Torbat Heydariyeh University of Medical Sciences, Torbat Heydariyeh, Iran; d Department of Infectious Diseases, Faculty of Medicine, Antimicrobial Resistance Research Center, Mazandaran University of Medical Sciences, Sari, Iran; e Toxoplasmosis Research Center, Communicable Diseases Institute, Department of Parasitology, Mazandaran University of Medical Sciences, Sari, Iran; f Department of Parasitology and Mycology, Faculty of Medicine, Mazandaran University of Medical Sciences, Sari, Iran; g Department of Pathobiology, Faculty of Veterinary Medicine, Bu-Ali Sina University, Hamedan 6517658978, Iran

**Keywords:** Hospital, Myiasis, Iran, Nosocomial, *Chrysomya bezziana*

## Abstract

*Chrysomya bezziana* can potentially cause myiasis, a condition in which fly larvae infest living tissue in humans and animals. Contributing factors to this rare ailment include poor oral hygiene, alcohol abuse, and infected wounds. Hospital-acquired myiasis, a rare type of myiasis, occurs in patients following hospital admission. Herein, we report a case of *C. bezziana* oral myiasis in an 89-year-old female patient hospitalized in northeastern Iran. In this article, we also present an updated review of reported human myiasis cases in Iran up to 2025. According to our findings, *C. bezziana* has been responsible for 13 documented cases of myiasis in Iran. Most nosocomial myiasis cases in the country are related to pharyngeal and nasal infestations and are primarily observed in the ICU patients.

## Graphical Abstract

**Figure:**
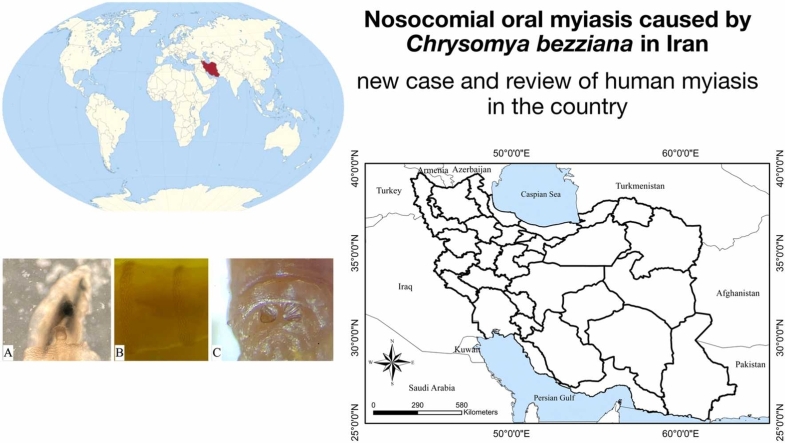

## Background

Myiasis is an opportunistic infestation of living tissue in vertebrates, with the larvae of dipteran flies belonging mainly to the families Oestridae, Calliphoridae, and Sarcophagidae. For oral myiasis, which is rarely reported, some contributing factors, including malocclusion, poor oral hygiene, tooth extraction, and halitosis, have been reported in the literature [1]. Moreover, in mentally disabled individuals and those with persistent mouth openings, the female flies to hosts with wounds or moist body openings where hundreds of eggs are laid [2]. Myiasis commonly affects the skin, eyes, nose, urogenital organs, stomach, and intestines, but can also affect any part of the body [3].

Due to the Old World screw-worm fly, *Chrysomya bezziana* (*C. bezziana*) Villeneuve, 1914 (Diptera: Calliphoridae), myiasis remains an important anthropozoonotic disease throughout much of Africa and Asia [4]. This obligate parasite belongs to the order Diptera, family Calliphoridae, suborder Cyclorrhapha, and poses significant losses to the livestock industry, especially in tropical regions [2], [5]. Human myiasis caused by *C. bezziana* was first reported in 1909 in India [6]. A 2019 systematic review article on human *C. bezziana* myiasis summarized 291 cases and found that these cases often occurred in patients with poor hygiene, low socioeconomic status, old age, and underlying diseases, including infections, age-related diseases, and noninfectious chronic diseases. The authors implied that *C. bezziana* myiasis is largely neglected, mainly as a serious medical or veterinary condition, with human and animal cases reported in only 16 and 24 countries, respectively, despite this fly species being recorded in 44 countries worldwide [2].

Herein, we report a case of *C. bezziana* oral myiasis in an 89-year-old female patient hospitalized in northeastern Iran. A comprehensive review of all reported cases of human myiasis in Iran up to 2025 is also presented, detailing the genus and species of the causative flies, patients’ age and gender, site of infection, reason for hospitalization, hospital department, and associated risk factors.

### Case presentation

In June 2024, an 89-year-old female with complaints of hypertension, dyspnea, and weakness presented to the Emergency Department of Imam Khomeini Hospital, Shahroud, Iran. Several months ago, the patient contracted COVID-19 before being admitted to the hospital. She was immediately transferred to the ICU due to poor general condition, low blood oxygen, and was intubated. The patient underwent laboratory examinations, and necessary measures were carried out (Table 1).

**Table 1:** Laboratory findings for the *Chrysomya bezziana*-infested patient.

**Tests**	**Value**	**Unit**	**Normal range**[7]
Total white cell count	9.9 × 10^9^	cells/L	4500–10,000
Hemoglobin	8.6	g/dL	12–16
Platelet	63 × 10^9^	cells/L	150–450
CRP	+ +	Qualitative	Negative
Troponin I	7488.0	ng/mL	< 0.01 ng/mL

On the 20th day post-hospitalization and during the oral examination, a bleeding ulcer was observed beneath the tongue of the patient, while she had no history of taking anticoagulant drugs or trauma to the mouth. Further examination revealed larvae of a myiasis-inducing fly. The larvae were collected and transferred to absolute ethanol in the Laboratory of Parasitology, Faculty of Veterinary Medicine, Bu-Ali Sina University, Hamedan, Iran, under cold chain conditions.

**Fig. 1:**
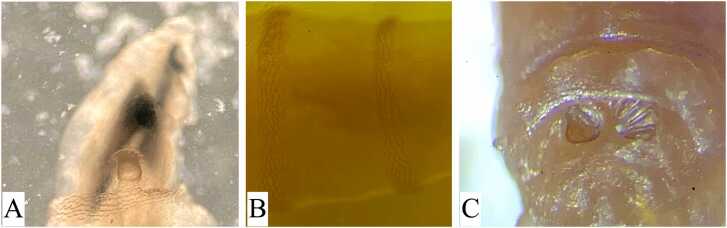
Morphology of the third-instar larva of *Chrysomya bezziana* collected from an 89-year-old female patient in Iran. A) Anterior part: anterior spiracle with a typical palmate shape due to six papillae arranged in a single row bearing oval spiracular openings. B) Midbody: intersegmental spines presented single, darkened, and tapered tips recurved toward the body. C) Caudal end: thick and incomplete, slightly convergent inner and middle spiracular slits and slightly divergent outer slits.

Morphologically, the anterior end showed the strong and robust mouth hook and the spiracle with a typical palmate shape due to six papillae arranged in a single row bearing oval spiracular openings (Fig. 1A). The intersegmental spines presented single, darkened and tapered tips recurved toward the body (Fig. 1B). In the caudal end, peritreme was thick and incomplete, dorsal ends of the inner and middle spiracular slits were slightly convergent; whereas, that of the outer slits was slightly divergent (Fig. 1C). The larvae were diagnosed as the third-instar larva of Old World screw-worm fly *C. bezziana* Villeneuve, 1914 (Diptera: Calliphoridae) according to a differential diagnosis key [8].

The patient underwent continuous mouth rinsing and was treated with a 150 µg/kg oral ivermectin tablet (Stromectol®, Merck Sharp & Dohme, Rahway, USA), an antiparasitic drug. Unfortunately, despite these efforts, the patient died because of cardiac arrest three days after the observation of oral myiasis.

### Literature review

A comprehensive search was conducted across international databases, including Google Scholar and PubMed, as well as Iranian databases such as IranDoc, Magiran, Iranmedex, and SID, to collect all reported cases of myiasis in English and Persian. All available literature on human myiasis cases in Iran through 2025 was collected (Table 2). In total, 120 patients aged between 7 days and 89 years, from 24 provinces, were reported across 75 publications presented as journal articles.

**Table 2:** Review of reported human myiasis cases in Iran until 2025.

**Type of myiasis**	**Agent**	**Sex / age**	**City (location in Iran)**	**Reason for referral**	**Inpatient ward**	**Nosocomial**	**Rick factor(s)**	**Reference**
Urogenital myiasis	*Megaselia scalaris*	Male / 18	Zanjan (Northwest)	Difficulty in urination, nocturnal enuresis	–	–	Lack of personal hygiene	[9]
	*Lucilia sericata* + *Wohlfahrtia magnifica*	Male / 86	Arak (Central)	Penile ulcer	–	–	Lack of personal hygiene	[10]
	*Psychoda* sp.	Female / 26	Kashan (Central)	Stomach pain, frequent urination, smelly and reddish vaginal discharge	–	–	Lack of personal hygiene	[11]
	*Lucilia sericata*	Male / 10 month	Isfahan (Central)	Restlessness, abdominal pain	–	–	Lived in rural areas	[12]
	*Psychoda albipennis*	Female / 9	Miandoab (Northwest)	Dysuria, several black-grayish colored mobile particles in his urine	–	–	Lack of personal hygiene	[13]
	*Megaselia scalaris*	Male / 60	Tehran (North-central)	Urinary tract problems	–	–	Travel to Thailand	[14]
	*Sarcophaga* sp.	Male / 7 day old	Ahvaz (Southwest)	Larvae in scrotum	Pediatric	–	Using unclean diapers, low socioeconomic status	[15]
	*Chrysomya bezziana*	Female / 36	Isfahan (Central)	Abdominal pain, dysuria	Emergency	–	Slight dysuria, a tingling feeling in the urethral region	[16]
	*Chrysomya bezziana*	Male / 5	Bushehr (South)	Persistent fever, significant weight loss	Pediatric	–	Lived in suburban areas, misdiagnosis of myiasis with enterobiasis	[17]
	*Sarcophaga* sp.	Female / 52	Kermanshah (West)	Persistent dysuria	Urology	–	Lack of personal hygiene, underlying urinary tract abnormalities	[18]
	Not stated	Female / 40	Kerman (Southeast)	Discharge, severe pain, swelling of the genital area	Gynecology	–	Low socioeconomic status, blindness	[19]
	*Wohlfahrtia magnifica*	Female / 20	Shiraz (South)	Pruritic lesion on vulva	–	–	Poor personal hygiene, swimming in the river	[20]
Ophthalmomyiasis	*Oestrus ovis*	Male / 35	Kerman (Southeast)	Sharp pain in the eyes	Emergency	–	Collecting material in the field	[21]
	*Lucilia sericata*	Male / 2	Tehran (North-central)	Conjunctival hyperemia, mild chemosis with obvious discomfort	–	–	Suicide, coma, ulcer	[22]
	*Lucilia sericata*	Male / 78	Kurdistan (Northwest)	Eye pain with mucoid ocular discharge, headache, sensing the presence of a foreign body in the eye, itchy eye	–	–	History of vascular tumor of the retina	[23]
	*Lucilia sericata*	Female / 80	Tehran (North-central)	Foreign body sensation in the right orbit and around the nose	Emergency	–	Invasive basal cell carcinoma, lesions in the right eye and removal of the right globe, diabetes mellitus	[24]
	Not stated	Male / 89	Tehran (North-central)	Progressive orbital mass	Oculoplastic	–	Lack of personal hygiene, history of surgery and cancer	[25]
	*Chrysomya bezziana*	Female / 55	Mazandaran (North)	Eye pain, extensive destruction of the left orbital cavity	–	–	Basal cell carcinoma	[26]
	*Chrysomya bezziana*	Male / 75	Tabriz (Northwest)	Eye pain, extension of the tumor into the orbit and globe of his eye	–	–	Periocular squamous cell carcinoma	[27]
	*Chrysomya bezziana*	Female / 87	Ahvaz (Southwest)	Eye pain, severely necrotized left orbit	–	–	Physical disability, history of eyelid surgery and cancer	[28]
	*Oestrus ovis*	Male / 34 (mean age for 4 cases)	Yazd (Central)	Acute conjunctivitis	–	–	History of contact with the flies	[29]
	*Oestrus ovis*	Male / 15 Male / 18 Male / 24 Male / 27 Male / 34 Male / 36 Male / 56 Female / 41	Fars (South)	Severe conjunctivitis, itchy eye, foreign body sensation	–	–	Lived in rural areas, close contact with sheep and goat	[30]
	*Oestrus ovis*	Female / 40	Mazandaran (North)	Conjunctivitis	–	–	Close contact with sheep and cow	[31]
	*Sarcophaga* sp.	Male / 62	Isfahan (Central)	Conjunctivitis	–	–	Basal cell carcinoma	[32]
	*Oestrus ovis*	Female / 38	Tehran (North-central)	Eye pain, itchy eye	Ophthalmology	–	History of contact with the fly	[33]
	*Chrysomya bezziana*	Male / 18	Lamerd (South)	Oral lesion, inability to feed orally	–	–	Congenital cerebral palsy, mental retardation, low economic status, poor hygiene	[34]
Oral myiasis	*Oestrus ovis*	Male / 3	Hamedan (West)	Gum bleeding, discomfort in chewing	–	–	Live close to livestock	[35]
	*Wohlfartia magnifica*	Male / 4	Bushehr (South)	Anorexia, weight loss	–	–	Mental retardation	[36]
	*Wohlfartia magnifica*	Male / 79	Kermanshah (West)	Toothache with a history of dizziness, headache and drowsiness for one week	–	–	Poor oral hygiene	[37]
	Calliphoridae	Male / 81 Male / 47 Female / 74	Tabriz (Northwest)	Respiratory distress, apnea, shortness of breath and chest pain	ICU	+	Poor oral hygiene, hemodialysis, intubation and mechanical ventilation in the ICU	[38]
	*Lucilia sericata*	Female / 78	Mazandaran (North)	Cardiac arrest	ICU	+	Intubation, presence of flies in the ICU	[39]
	Not stated	Male / 28	Tehran (North-central)	Itching and extreme discomfort on the entire maxillary and mandibular gingivae	–	–	Poor oral hygiene	[40]
	Not stated	Male / 89	Not stated	Tachypnea, tachycardia, decreased level of consciousness	–	–	Dementia, non-Hodgkin lymphoma and mechanical ventilation	[41]
	*Wohlfartia* sp.	Female / 8	Tehran (North-central)	Oral erythema, edema	–	–	Poor oral hygiene	[42]
Pharyngeal myiasis	*Oestrus ovis*	32male and 1 female / 11–62 years	Fars (South)	Foreign bodies sensation followed by burning and itching in the throat, cough	–	–	Close contact with sheep and goats	[43]
	*Oestrus ovis*	Male / 55	Western Azerbaijan (Northwest)	Respiratory distress, chronic obstructive pulmonary disease (COPD)	ICU	+	Addiction, nasogastric intubation with a mechanical ventilator	[44]
	*Lucilia sericata*	Female / 36	Zanjan (Northwest)	Progressive respiratory distress	ICU	+	Coma, intubation	[45]
	*Lucilia sericata*	Male / 63	Tehran (North-central)	Heart failure, coronary artery and mitral valve replacement	–	–	Long-term treatment with antibiotics and steroids, severe respiratory and heart failure	[46]
	*Lucilia sericata*	Male / 18	Kurdistan (Northwest)	Accident sustaining head trauma	ICU	+	Coma, intubation, COVID-19	[47]
		Male / 32				+	Addiction, coma, intubation	
	*Wohlfartia nuba*	Female / 5.5	Golestan (North)	Heart surgery	ICU	+	Hospitalized in the ICU	[48]
	Not stated	Female / 52	Bojnord (Northeast)	Weakness, nausea, vomiting	ICU	+	Diabetes mellitus, intubation	[49]
Nasal myiasis	*Lucilia sericata*	Male / 69	Babol (North)	Dyspnea, stridor, coughing, nasal discharge, digestive problems	Emergency	+	Chronic pulmonary problems, lack of awareness	[50]
	*Lucilia sericata*	Female / 50	Kermanshah (West)	Nasal discharge, coughing	Psychiatry	+	Being in contact with livestock and pets	[51]
	*Lucilia sericata*	Female / 12	Tehran (North-central)	Cerebral palsy (CP), sepsis, respiratory distress, hypoglycemia	ICU	+	Decreased level of consciousness, intubation, broad-spectrum antibiotics consumption	[52]
	*Lucilia sericata*	Male / 74	Mazandaran (North)	Cardiac arrest	CCU	–	Coma, diabetes mellitus	[53]
	*Lucilia* sp.	Male / 35	Ahvaz (Southwest)	Respiratory distress, loss of consciousness	ICU	+	Gastric cancer, chemotherapy	[54]
	*Lucilia sericata*	Female / 54	Tehran (North-central)	Coronary artery bypass grafting	ICU	+	Intubation and mechanical ventilation	[55]
	*Chrysomya bezziana*	Female / 74	Gonabad (Northeast)	Respiratory distress, exertional dyspnea, fever	ICU	+	Broad-spectrum antibiotics consumption, loss of consciousness, intubation, sepsis	[56]
	*Chrysomya bezziana*	Male / 45	Iranshahr (Southeast)	Headache, vertigo, epistaxis, facial edema	–	–	Exposed to insects on the farm	[57]
	*Eristalis tenax*	Female / 14	Tehran (North-central)	Coryza, nasal grip, mild dyspnea, coughing	–	–	Living in a rural area, taking antibiotics, and corticosteroids	[58]
	Not stated	Male / 63	Tehran (North-central)	Epistaxis, nasal obstruction, nasal discharge, the presence of larvae	–	–	Diabetes mellitus, hypertension, and kidney transplantation	[59]
	*Sarcophaga argyrostoma*	Female / 32	Golestan (North)	Fever, gastrointestinal pain, cramps, vomiting, weight loss, fatigue	–	–	Immune deficiency, kidney transplantation, intestinal cytomegalovirus	[60]
	*Sarcophaga* *fertoni*	Female / 7	Sanandaj (West)	The presence of larvae in the stool	-	–	Poor personal hygiene	[61]
	*Sarcophaga* *haemorrhoidalis*	Male / 13	Kuhrang (Central)	Abdominal pain, feeling of filled stomach, loose stool (2–3 times per day), weight loss	-	–	Living in a rural area, poor hygiene	[62]
Gastrointestinal myiasis	*Sarcophaga* sp*.*	Male / 34	Kurdistan (West)	Abdominal distress, gastroenteritis, abdominal pain, loose feces	–	–	Working in the stable	[63]
	*Lucilia illustris*	Female / 45	Kurdistan (West)	Abdominal pain, loose stool	–	–	Living in a rural area	[64]
	*Eristalis tenax*	Female / 22	Babol (North)	The presence of larvae in feces	–	–	Poor personal hygiene	[65]
	*Eristalis tenax*	Female / 4	Bajestan (Northeast)	Anal itching, one live larva in feces	–	–	Living in a rural area, consuming subterranean water	[66]
Scalp myiasis	*Chrysomya bezziana*	Male / 5	Hormozgan (South)	Severe headache, agitation symptoms	Pediatric	–	Open wound, poor personal hygiene, being in contact with goats	[67]
	*Chrysomya bezziana*	Male / 56	Arak (Central)	A mass on the scalp	–	–	Living in a rural area	[68]
	Not stated	Female / 64	Isfahan (Central)	A large ulcerative, hemorrhagic area on the scalp	–	–	Basal cell carcinoma	[69]
	*Sarcophaga* sp.	Male / 43	Qazvin (North)	Progressive scalp ulceration	–	–	Soft tissue sarcoma	[70]
	*Lucilia sericata*	Male / 22	Tehran (North-central)	Itching sensation within the scalp ulcer	Dermatology	-	Suicide, coma and ulcer	[22]
Wound myiasis	*Lucilia sericata*	Female / 62	Khuzestan (Southwest)	Coronary artery bypass grafting (CABG)	CCU	+	Diabetes mellitus, non-healing wound in mandible	[71]
	*Lucilia sericata*	Male / 62	Tehran (North-central)	Abnormally growing and ruptured neck mass containing worms	–	–	Being homeless, low socioeconomic status, crack cocaine consumption	[72]
	*Lucilia sericata*	Male / 26	Kashan (Central)	Hypoxic encephalopathy and coma	–	–	Addiction (heroin, opium), HCV positive	[73]
	*Chrysomya bezziana*	Male / 87	Shiraz (South)	Severe itching of both lower extremities	–	–	Diabetes mellitus, addiction, cardiovascular disease	[34]
	*Chrysomya bezziana*	Female / 3	Hormozgan (South)	Ingestion of the aluminum hydroxide powder	Pediatric	–	Open wound, lived near livestock in a rural area	[74]
	*Cordylobia anthropophaga*	Male / 40	Isfahan (Central)	Several red pruritic popular lesions on thighs	–	–	Travel to Africa	[75]
	*Calliphora vicina*	Female / 11 month	Hamedan (West)	Buccal swelling, erythema	–	–	Buccal ulcer - 11 month-old infant	[76]
	*Calliphora* sp.	Male / 70	Tehran (North-central)	Bilateral plantar ulcers	–	–	Diabetes mellitus	[77]
	*Lucilia cuprina*	Male / 85	Ardabil (Northwest)	Infection of the thumb finger of the left foot	Infection	–	Diabetes mellitus, blindness	[78]
	*Lucilia sericata*	Male / 63	Mashhad (Northeast)	Progressive lung cancer, apnea	ICU	+	Coma, tracheotomy intubation, open wound	[79]
Otomyiasis	*Chrysomya bezziana*	Male / 55	Kashan (Central)	Ear discharge, ear pain	ENT	–	Poor hygiene, working in contaminated areas	[80]
	*Lucilia sericata*	Female / 62	Ahvaz (Southwest)	Edema and decompensated heart failure, intense swollen and erythematous right ear	ICU	+	Intense swollen and erythematous right ear	[81]

## Discussion

The maggots identified herein were the larvae of *C. bezziana*, a fly species widely distributed in the West, South, and East of Asia (*e.g*., Iran, India, Saudi Arabia, Indonesia, Papua) and Africa. The first reported human infection by these flies was documented in Algeria in 1997 by Abed-Benamara [23], [82]. Before this study, only one case of oral myiasis caused by *C. bezziana* was reported in southern Iran; the patient suffered from congenital cerebral palsy and was in poor health and economic conditions [34]. In Iran, *C. bezziana* is a well-known parasitic threat to livestock, particularly affecting cattle, sheep, goats, and camels. Its larvae infiltrate wounds or natural body openings, resulting in severe tissue damage, secondary infections, and, in extreme cases, systemic toxicity. Furthermore, studies have shown that livestock herds in southern Iran are especially at risk, with infestations leading to significant economic losses. These include reduced productivity, increased veterinary expenses, and, occasionally, fatalities among infected animals [83], [84].

As summarized in Table 2, a total of 120 cases of human myiasis were recorded in Iran by 2025. Most patients resided in the northern and central regions of the country. The predominant causative agents of myiasis in Iran were *Oestrus ovis*, *Lucilia sericata*, and *Chrysomya bezziana*. Various types of myiasis have been reported, including pharyngeal (40 cases), ocular (23), cutaneous (15), urogenital (12), oral (11), nasal (10), gastrointestinal (7), and auricular (2) cases.

Oral myiasis is a rare medical condition linked to poor oral hygiene, excessive alcohol consumption, advanced age, and wounds exposed to harmful microorganisms [9]. This myiasis manifests with symptoms such as gum inflammation, oral mucosal injury, gum necrosis, varying degrees of pain, and oozing lesions [16]. Oral myiasis has been observed in severely diseased individuals on mechanical ventilation in previous studies [10], [38]. Flies responsible for oral myiasis in Iran included *Oestrus ovis*, *Wohlfahrtia magnifica*, *C. bezziana,* and *Lucilia sericata* (Table 2). In the current study, the patient was elderly, had a history of tooth extractions, was on mechanical ventilation, and slept with his mouth partially open, which increased his vulnerability. Oral myiasis caused by *C. bezziana* was also observed in an 18-year-old male with cerebral palsy, cognitive impairment, and quadriplegia in Fars Province, Iran [34].

Furthermore, three additional cases of *C. bezziana* oral myiasis were reported in patients hospitalized in the ICU in Tabriz, northwest Iran. These patients were intubated, on mechanical ventilation, and had poor oral hygiene, which increased their risk of developing oral myiasis [38]. During the spring and summer months, warmer temperatures and increased humidity create ideal conditions for flies to lay eggs on the skin or in open wounds, leading to myiasis [85]. In the reports on nosocomial myiasis in Iran, the most common risk factors for its occurrence include intubation, immune system diseases like diabetes and cancer, coma and unconsciousness, open wounds, blindness, addiction, and the presence of myiasis-producing flies in various hospital wards. While reports of nosocomial myiasis in Iran are relatively scarce, its significance should not be underestimated. Essentially, the presence of such infestations in hospitals indicates improper management practices.

Here, we report the 20th reported nosocomial myiasis case in Iran, where the majority of patients were diagnosed in the ICUs (Table 2). Of the four documented cases of nosocomial oral myiasis reported so far in Iran, all cases occurred in the ICU patients [38], [39]. Nosocomial myiasis occurs when a patient becomes infested after hospital admission, making prevention a priority for hospital authorities [26]. In low-income and developing countries, nosocomial myiasis highlights the need for increased awareness and improved medical infrastructure [27]. Hospital myiasis not only prolongs hospitalization but also delays treatment of the disease. Preventive measures such as proper wound care, maintaining hygiene, and educating healthcare workers about the risks of myiasis are essential to reduce its incidence. Early recognition and treatment can significantly improve outcomes and reduce complications [3], [25].

There is no definitive evidence linking parasitic infections to increased severity of COVID-19. However, the use of immunosuppressive medication in COVID-19 patients may raise the risk of severe parasitic infections. The first recorded case of nosocomial myiasis along with COVID-19 occurred in 2021 in an elderly male patient hospitalized in the ICU in Serbia. The patient exhibited high fever, respiratory distress, weakness, general discomfort, nausea, and a history of autoimmune disorders [11]. In 2023, another case of nosocomial myiasis in the nasal area was reported in the ICU in Iran, associated with COVID-19 [47]. Our case represents the second nosocomial myiasis in a COVID-19 patient, this time involving the oral cavity. In both patients, intubation and loss of consciousness were identified as risk factors for contamination.

## Conclusion

Hospitalized individuals, particularly those in the ICU, require extensive care, with particular attention to oral hygiene. In addition to practicing good hand and oral hygiene, it is essential to maintain a clean environment by installing nets on windows, ensuring adequate ventilation, refraining from opening ward windows, and using insect management measures to eliminate flies. Moreover, healthcare workers should have the necessary information about this parasite and pay more attention to people at risk. Following specific care protocols, including safety guidelines and improving quality standards, can help reduce costs and prevent adverse consequences for patients, including those in the intensive care unit.

## Data availability

Data is provided within the manuscript. Any additional data is available from the corresponding author on request.

## CRediT authorship contribution statement

**Eissa Soleymani:** Supervision, Data curation. **Fatemeh Nikbin:** Validation, Software, Conceptualization. **Alireza Sazmand:** Writing – review & editing, Methodology, Investigation. **Mohammad Ali Mohaghegh:** Validation, Software, Conceptualization. **Fateme Skandary:** Writing – original draft. **Seyed Reza Mirbadie:** Investigation, Formal analysis, Conceptualization.

## Consent for publication

Written informed consent for publication of the patient’s details was obtained from her next of kin.

## Ethics approval and consent to participate

The research was approved by the Ethics Committee of Shahrood University of Medical Sciences under the code: IR.SHMU.REC.1403.157.

## Funding

No funding was used in this study.

## Declaration of Competing Interest

We have no conflicts of interest to disclose. All authors declare that they have no conflicts of interest.
